# The Circadian Binding of CLOCK Protein to the Promoter of *C/ebpα* Gene in Mouse Cells

**DOI:** 10.1371/journal.pone.0058221

**Published:** 2013-03-11

**Authors:** Haruhisa Kawasaki, Ryosuke Doi, Kumpei Ito, Masami Shimoda, Norio Ishida

**Affiliations:** 1 Ishida Group of Clock Gene, Biomedical Research Institute, National Institute of Advanced Science and Technology (AIST) 6-5 Central, Tsukuba, Ibaraki, Japan; 2 Graduate School of Life and Environmental Sciences, Tsukuba University, Tsukuba, Ibaraki, Japan; 3 Division of Cell Physiology, Department of Physiology and Cell Biology, Graduate School of Medicine, Kobe University, Kobe, Hyogo, Japan; 4 Division of Insect Sciences, National Institute of Agrobiological Science, Tsukuba, Ibaraki, Japan; Pennsylvania State University, United States of America

## Abstract

C/EBPα plays important roles in metabolism as well as in the maintenance of energy homeostasis. Here we describe loss of the circadian oscillation of *C/ebpα* expression in liver of *Clock* mutant mice. Reporter assays indicate Clock and Bmal significantly induced *C/ebpα* gene expression whereas Cry suppressed. Real time reporter assays showed that two mutated E-boxes disrupted *C/ebpα* promoter dependent-oscillation. Chromatin immunoprecipitation suggests Clock can bind to two E-boxes in the *C/ebpα* promoter with a circadian manner *in vivo*. Thus, *C/ebpα* gene transcription is under circadian control of a core clock component, *Clock*. The data suggests that circadian disturbances may affect metabolic abnormalities through the *C/ebpα* pathway in liver.

## Introduction

Many organisms display physiological and behavioral rhythms of entrainment to a 24-h cycle of light and darkness. The master clock located in the suprachiasmatic nucleus of mammals controls most physical and physiological circadian rhythmicity [1], [2] and the generation of circadian rhythms depends on the concerted co-expression of specific clock genes. Clock was the first clock gene identified in vertebrates [3]. CLOCK binds DNA and activates transcription after dimerization with BMAL1 [1], [2] by driving the rhythmic transcription of other clock and circadian clock-controlled genes through an E-box (CACGTG). Because the Clock allele of Clock mutant mice is truncated and causes a deletion of 51 amino acids, the mutation presumably would not significantly affect N-terminal basic helix-loop-helix and Per-Arnt-Sim domains, leaving CLOCK dimerization and DNA binding intact. The mutant CLOCK protein can still form heterodimers with BMAL1 that binds to DNA, but these heterodimers are deficient during transactivation [2]. The mammalian circadian clock is an intracellular, transcriptional-translational mechanism comprising the same molecular components in the suprachiasmatic nucleus and in peripheral cells. Because this endogenous timekeeper interacts with countless biological systems, circadian disruption has significant effects on health; for example, susceptibility to obesity, diabetes and related metabolic syndromes and various types of cancer is increased among long-term shift workers [4].

The founder of the family of related leucine zipper transcription factors is CCAAT enhancer binding protein alpha (C/EBPα), which plays an important role in numerous cellular processes including proliferation, differentiation, apoptosis, metabolic control and other specific functions [5]–[8]. This gene is expressed at high levels in the liver and it is critical for the establishment and maintenance of energy homeostasis in neonates [9], [10]. *C/ebpα* gene knockout mice die because they cannot accumulate glycogen in the liver and develop hypoglycemia [10].

Hundreds of tissue-specific circadian genes that regulate an impressive diversity of biological processes have been identified using DNA microarray technology [11]–[14], and we found that *C/ebpα* is one of circadian expressing gene candidates that have been screened from Database of Circadian Gene Expression (http://expression.gnf.org/cgi-bin/circadian/index.cgi) and Database for Systems Biology (http://sirius.cdb.riken.jp/). Some studies have shown that mutations or deletions of *Clock* and *Bmal1* genes result in not only circadian disturbances but also disrupted glucose and lipid metabolism [15]–[18]. In fact, recent reports indicate that C/EBPα directly controls *Per2* and *Rev-Erbα*, which are core members of clock genes for the circadian clock [19], [20]. However, little is known about the molecular mechanism of circadian oscillation of *C/ebpα* gene expression.

We identified E-boxes in the *C/ebpα* promoter region investigated the relationship between *C/ebpα* and clock genes. Here, we show that the core clock gene product CLOCK regulates the circadian expression of *C/ebpα* gene in mouse cells.

## Materials and Methods

### Ethics Statement

All animal experiments, care and handling proceeded under the approval of our institutional Animal Care and Use Committee (Permission Number 2009–020).

### Animals

Male Jcl:ICR (Clea Japan Inc., Tokyo, Japan) and homozygous Clock mutant mice on a Jcl:ICR background [21] aged 7–10 weeks were maintained under a 12 h light/12 h dark cycle (lights on at 0∶00 and lights off at 12∶00) for at least two weeks before experimentation. The mice were fasted overnight and sequentially sacrificed for some experiments.

### Isolation of mRNA and RT-PCR

Total RNA was isolated from NIH 3T3 cells or liver tissue using RNAiso (TAKARA Bio Inc., Shiga, Japan) and then reverse-transcribed using the PrimeScript RT reagent kit (TAKARA Bio) according to the manufacturer’s protocol. 3 animals were used per each time. The cDNA levels of genes of interest were measured by real time quantitative PCR using a LightCycler (Roche Applied Science) with SYBR Premix Ex Taq (TAKARA Bio). The amount of mRNA was corrected relative to that of *β-actin* for liver tissue or *Large Ribosome Protein* (*Rplpo*) for NIH 3T3 cells. The maximal value for wild type mice is expressed as 100% and other values are expressed means ±SEM (n = 3).

### Cell Culture

NIH3T3 cells were incubated in Dulbecco’s modified Eagle’s medium supplemented with 10% fetal bovine serum and a mixture of penicillin and streptomycin at 37°C under a humidified 5% CO_2_ atmosphere.

### Transient Luciferase Assays

The upstream region of the *C/ebpα* transcription start site containing two (−1386 to +113 bp) E-boxes were cloned into the pGL3 Basic vector (Promega). Mouse CLOCK, BMAL1, and CRY1 expression plasmids were provided by Dr. T. Todo [22]. Constructs (500 ng) were co-transfected with 1 ng of pRL-CMV (Promega) as the internal control into NIH3T3 cells (24-well plates) using HilyMax (DOJINDO Laboratories, Kumamoto, Japan) according to the manufacturer’s protocols. Luciferase activities were measured using a dual luciferase reporter assay system (Promega) and a Luminometer model TD-20/20 (Turner Designs, Sunnyvale, CA). The transcriptional activities were normalized relative to Renilla luciferase activities.

### Real time Luciferase Assays

The upstream region of the *C/ebpα* transcription start site containing two E-boxes (−1386 to +113 bp) was cloned into SV40-dLuc harboring the SV-40 promoter and a rapid degradation domain modified from mouse ornithine decarboxylase at the C-terminal end of firefly luciferase [13]. The *Per2* promoter regions (−798 to +331 relative to the cap site) were cloned into *pGL3-dLuc* [23], and then 2 µg of reporter plasmids were transfected into NIH3T3 cells (35-mm collagen type I-coated dishes) using HilyMax (DOJINDO Laboratories, Kumamoto, Japan). The cells were stimulated with 100 nM dexamethasone (Sigma-Aldrich) for 2 h in serum-free Dulbecco’s modified Eagle’s medium (D-MEM) and then the medium was replaced with fresh Dulbecco’s MEM containing 100 µM luciferin (Wako Pure Chemical Industries), 25 mM HEPES (pH 7.2), and 10% fetal bovine serum. Bioluminescence was measured and integrated for 1 min at intervals of 10 min using the Photon Detection UNIT LM-2400 (Hamamatsu Photonics, Hamamatsu, Japan). To compare the phase and amplitude of *C/ebpα -WT-dLuc* and each mutant, the data were detrended by subtracting an average of 12 h from the data.

### Chromatin Immunoprecipitation (ChIP) Assays

Immunoprecipitation was adapted from the reported procedure [24], [25]. NIH 3T3 cells were stimulated with dexamethasone and incubated for 10 min at room temperature. Formaldehyde was added directly to cell culture media at a final concentration of 1% at 0, 6, 12, 18, 24 or 30 h thereafter. Cross-linking was stopped by adding 125 mM glycine. The plates were rinsed with cold PBS and then NIH 3T3 cells harvested by scraping in 0.4 mL of ice cold 10 mM PMSF in PBS were sedimented by centrifugation. Binding between CLOCK and E-boxes was detected using the Simple ChIP Enzymatic Chromatin IP Kit (Cell Signaling Technology, Danvers, MA, USA) with anti-CLOCK antibody [26](SC-6927; Santa Cruz Biotechnologies, CA, USA) according to the product insert. Sample DNA was isolated from the immunoprecipitates and then amplified by PCR using the following primer sets: E1 site (from −1240 to −1128), 5′-GAGGGTGAACGAGACGCCACGTGGCTGGGAGCCGATGC-3′ and 5′-GCCTTTCGAGCACCCACTTGGG- 3′; E2 site (from −295 to −137), 5′-GCCTAACCACGGACCACGTGTGTGCGGGGGCGACAGCG-3′ and 5′-TGACTTTCCAAGGCGGTGAGTGGG-3′; E-box unrelated sequence (from −2239 to −2063), 5′-TGACCGGTTCCACCTCTAAC-3′ and 5′-CAGGGGTTCGTGAGAAATGT-3′; and the most proximal E-box of *Dbp* [27], [28], 5′-ACACCCGCATCCGATAGC-3′ and 5′- CCACTTCGGGCCAATGAG-3′.

### Statistical Analysis

All data are expressed as means ± SEM. Differences in expression levels and peak times were statistically evaluated using Student’s t-test for single comparisons and one-way ANOVA with post-hoc Student’s *t*-test for multiple comparisons.

## Results

### Oscillatory Expression of *C/ebpα* mRNA in Mouse Liver

**Figure 1:**
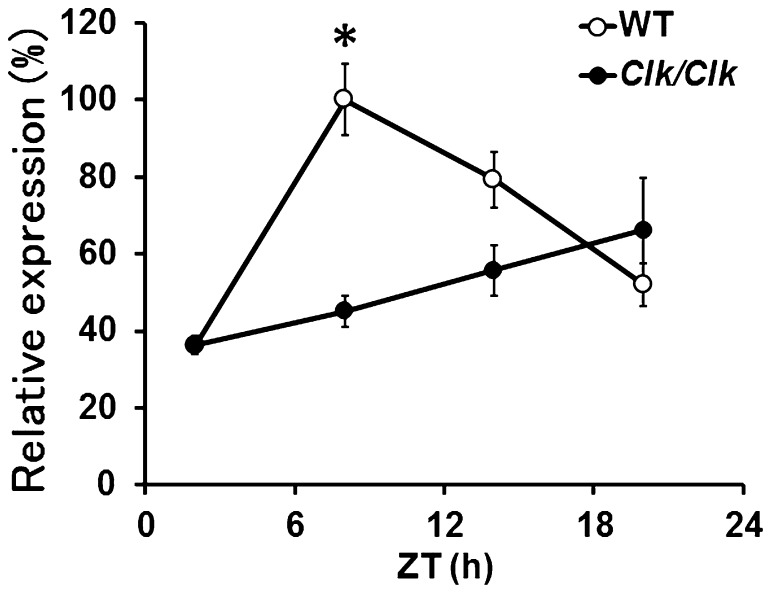
Clock mutation damps daily variation in *C/ebpα* mRNA expression. Total RNA was extracted from the livers of three wild-type (WT; open symbols) and three *Clock* mutant (*Clk*; closed symbols) mice maintained under a 12 h light/12 h dark cycle (lights on at 0∶00 and lights off at 12∶00) and then *C/ebpα* expression was determined by RT-PCR. Maximal value for wild-type mice is expressed as 100%. Each time point comes from average of three mice. Data are presented as mean ± SEM (n = 3, *: p<0.01).

Since *C/ebpα* is essential for liver development, we isolated *C/ebpα* mRNA from livers of wild-type and *Clock* mutant mouse to determine the relationship between the circadian clock and temporal *C/ebpα* gene expression using RT-PCR (Fig. 1). Diurnal expression of *C/ebpα* mRNA was robust in the liver of wild-type mice, but rhythmic expression was significantly dampened in those from *Clock* mutant mice. The *C/ebpα* mRNA levels obviously peaked at Zeitgeber time (ZT) 8 in the wild type liver.

### CLOCK Up-regulates *C/ebpα* Expression in vitro

**Figure 2:**
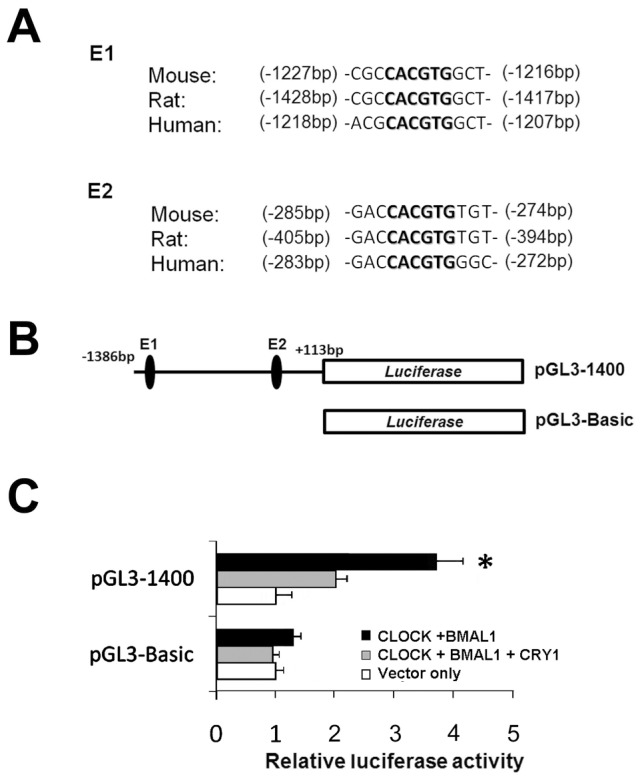
CLOCK and BMAL1 up-regulate the expression of *C/ebpα* promoter. (A) Comparison of mouse rat and human *C/ebpα* E-boxes. *C/ebpα* promoter contains two putative E-boxes within 1.4 kb region upstream of transcription start site. Consensus E-box sequences are shown in bold type. Positions from transcription start sites are shown in parentheses. This figure is based on information derived from the Mammalian Promoter/Enhancer Database (http://promoter.cdb.riken.jp/). (B) Schematic representation of reporter gene and constructs of *C/ebpα*promoter region. Plasmid *pGL3-1400* contains 1.4 kb upstream region of the *C/ebpα* transcription start site. Plasmid *pGL3-BASIC* does not contain *C/ebpα* promoter region. (C) Reporter gene assay for *C/ebpα* promoter. CLOCK, BMAL1 and CRY1 are overexpressed with the reporter constructs in NIH 3T3. *pGL3-1400* reporter was up-regulated by CLOCK and BMAL1 then CRY1 supressed the expression. Each column comes from triplicate experiments. Data are presented as mean ± SEM (n = 3, *: p<0.01).

Analysis of the mouse genome revealed two perfect E-box motifs within 1.4 kb of the 5′-flanking region of the *C/ebpα* gene (Fig. 2A), suggesting that clock gene products may control *C/ebpα*. We therefore analyzed the functions of these E-box elements using a genomic DNA sequence of this region fused to the luciferase reporter plasmid (Fig. 2B). The promoter reporter assay showed that the transcriptional activity of a 1.4 kb fragment containing the two E-boxes was increased 3-fold under CLOCK and BMAL1 overexpression in NIH 3T3 cells (Fig. 2C). This increase was suppressed by co-expression with CRY1, which is the negative component of CLOCK-BMAL1-dependent transcriptional activation [1]. The findings suggest that a feedback loop of the molecular circadian clock may regulate the upstream region of *C/ebpα* containing these two E-boxes.

### Upstream E1 and E2 Sites Regulate the Circadian Expression of *C/ebpα* Gene

**Figure 3:**
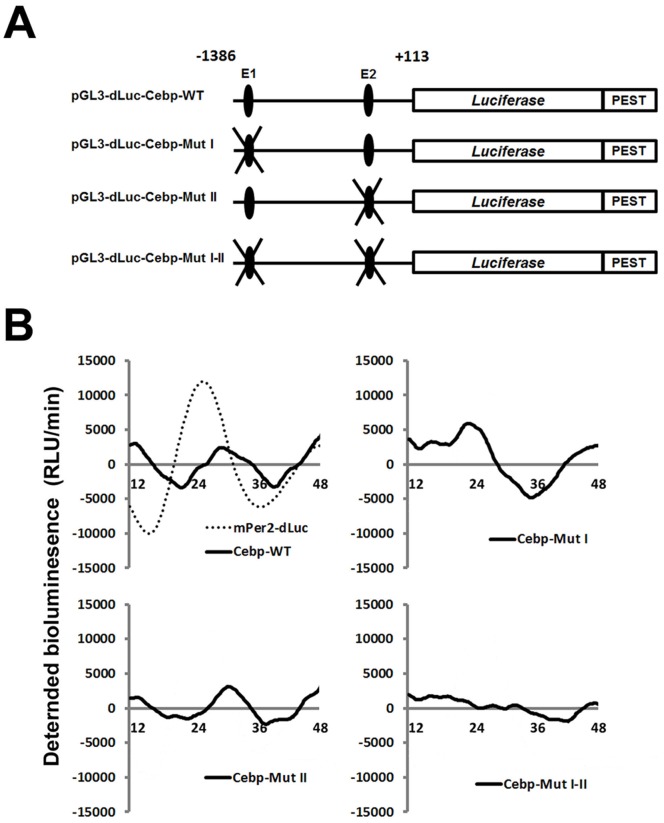
E1 and E2 sites contribute to circadian expression of *C/ebpα*. (A) Schematic representation of mutant constructs of *C/ebpα* promoter region. Either or both E-boxes (sites E1 and E2) were mutated (E1, 5′-CACGTG-3′ to 5′-CACCAC-3′; E2, 5′-CACGTG-3′ to 5′-CACCAC-3′). (B) Real time reporter assay. NIH3T3 cells transfected with indicated mutant constructs were incubated with dexamethasone (100 nM) and then bioluminescence was measured. Plasmid *pGL3-dLuc-Cebp-WT*, wild type 1.4 kb upstream region of *C/ebpα* gene;, *pGL3-dLuc-Cebp-Mut I*, mutant E1 site; *pGL3-dLuc-Cebp-Mut II*, mutant E2 site; *pGL3-dLuc-Cebp-Mut I-II*, mutant E1 and E2 sites; *mPer2-dLuc*, Per2 promoter region (−798 to +331 relative to the cap site) cloned into *pGL3-dLuc* [23]. Each experiment was repeated 3-5 times and representative data were used for these graphs.

We analyzed the roles of the two E-boxes in circadian expression of the *C/ebpα* gene using real time reporter assays with NIH3T3 cells containing functional circadian clock components. The transcriptional activity of the construct containing two E-boxes (Fig. 3A; *pGL3-dLuc-Cebp-WT*) exhibited circadian oscillation (Fig. 3B; Left upper). When E1 or E2 site was mutated (Fig. 3A; *pGL3-dLuc-Cebp-Mut I*, *pGL3-dLuc-Cebp-Mut II*), circadianoscillations of the reporter activity was still observed although with abnormal phase when E1 or E2 site was mutated (Fig. 3B; Upper right and Left lower). In contrast, mutations in both of E-boxes (Fig. 3A; *pGL3-dLuc-Cebp-Mut I-II*) disrupted the circadian oscillation of the reporter activity (Fig. 3B; Lower right). These findings show that both of the E1 and E2 sites are involved in circadian expression of the *C/ebpα* reporter gene in NIH3T3 cells.

### CLOCK Binds to E-boxes in *C/ebpα* Control Region in Living Cells

**Figure 4:**
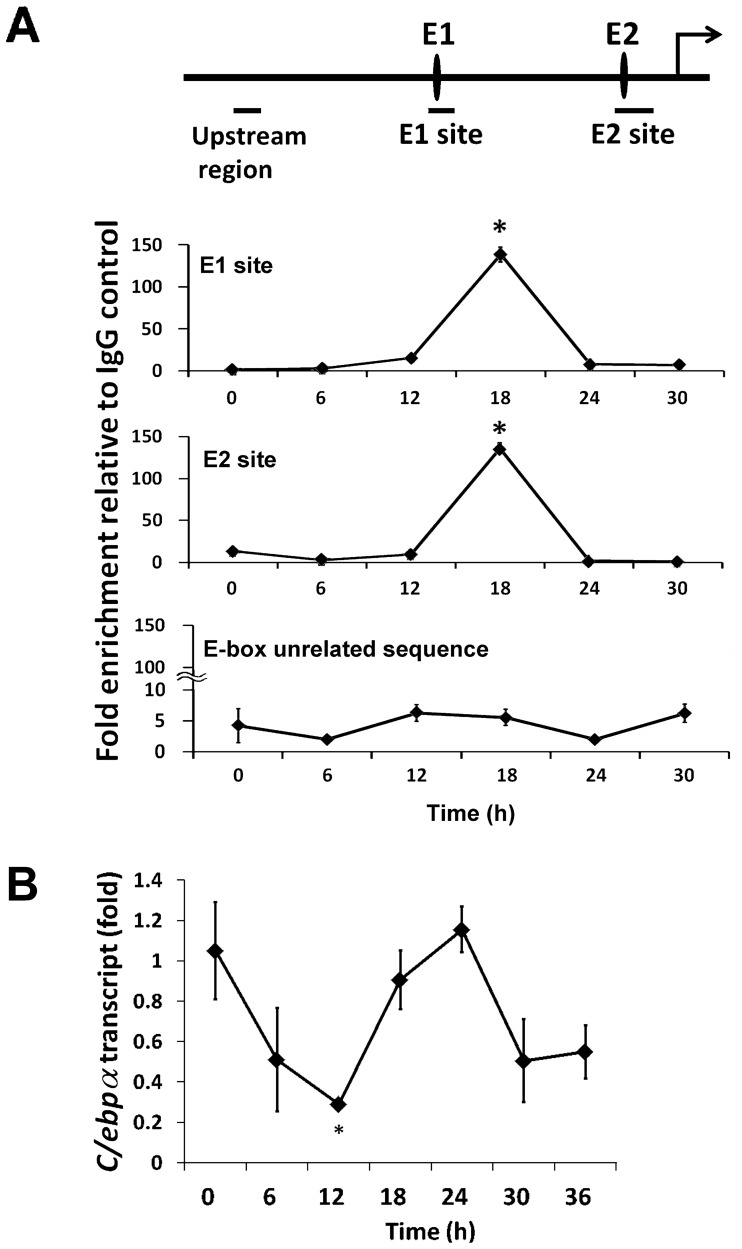
CLOCK binds to E1 and E2 sites in living cell. (A) Chromatin immunoprecipitation (ChIP) assay for CLOCK and E-boxes of *C/ebpα* promoter. E-box sites are shown as ovals on the schematic. Horizontal bars indicate amplified regions (E-box unrelated sequence, from −2239 to −2063; E1 site, from −1240 to −1128; E2 site, from −295 to −137.) NIH 3T3 cells were rapidly sampled and processed every 6 hours after dexamethasone stimulation. Chromatin immunoprecipitation was performed using antibodies against CLOCK, and enrichment of E-boxes containing sequence of the promoter region of *C/ebpα* were measured using quantitative PCR. Data were normalized to a control condition (Normal Rabbit IgG). The ChIP assay was repeated two times. Each time point comes from triplicate experiments. Data are presented as mean ± SEM (n = 3, *: p<0.0001). (B) Temporal mRNA expression of endogenous *C/ebpα* in NIH 3T3.Total RNA was extracted every 6 hours after dexamethasone stimulation and mRNA transcripts were quantitated relative to *Rplpo* transcript expression (housekeeping gene) by using quantitative RT-PCR. Each time point comes from triplicate experiments. Data are presented as mean ± SEM (n = 3, *: p<0.05).

We analyzed *in vivo* binding of CLOCK for the putative DNA sequence using chromatin immunoprecipitation (ChIP) assays in NIH 3T3 cells. To confirm the validity of this anti-CLOCK antibody for ChIP in 3T3 cells, we evaluated CLOCK binding to-E box element in *Dbp* (*D site albumin promoter binding protein*) as a positive control (Fig. S1). We found that CLOCK bound to both fragments of the *C/ebpα* 5′-flanking region that contain E-boxes (Fig. 4A). CLOCK binding to two E-boxes was detected in a circadian manner and peaked at 18 hour after dexamethasone treatment. In contrast, E-box unrelated sequence in upstream region has consistently detected very low binding with CLOCK (Negative control). We also determined temporal expression of endogenous *C/ebpα* by using RT-PCR and confirmed its circadian oscillation (Fig. 4B). These findings indicate that CLOCK protein bind to two E-boxes of the *C/ebpα* promoter in NIH 3T3 cells at the chromatin level and suggest that CLOCK controls the circadian expression of *C/ebpα* gene through these two E-boxes even in living cells.

## Discussion

Here, we discovered that the circadian expression of *C/ebpα* gene was regulated by two E-boxes in the upstream region of this gene through the core clock protein, CLOCK. Considering with transient luciferase assays, real-time reporter assays in vitro, and ChIP assays and loss of circadian mRNA expression in *Clock* mutant mouse liver, the data indicates that the molecular circadian feedback loop affects *C/ebpα* gene circadian expression through two E-boxes. *C/ebpα* protein is thought to play important roles in numerous cellular processes including cell proliferation, differentiation and apoptosis [5]–[8]. But, *C/ebpα* gene is expressed at high levels in the liver and recent reports show that it is critical for the establishment and maintenance of energy homeostasis in mammals [9]–[10]. Furthermore, the core clock proteins are reported to affect energy homeostasis in many species [29]. Genome-wide screening of BMAL1 and CLOCK targets has confirmed that carbohydrate and lipid metabolism comprises the major output of the circadian clock in the mouse liver [14], [30], [31].

Duplicate E-boxes were reported to be important for the circadian rhythmic mRNA expression of clock-controlled genes [30], [32]. We also found two E-boxes are required to generate the distinct circadian oscillation of *Gys2* (*Glycogen synthase 2*) gene expression in mouse liver [25]. These papers suggest that two E-boxes system are important for the circadian expression of clock-controlled genes in peripheral tissues. In this paper, ChIP assay and real time reporter assay indicated that the binding peak of CLOCK to E-boxes preceded the peak expression of endogenous *C/ebpα* in NIH 3T3 cell. This suggests that CLOCK binding *in vivo* might occur earlier than transcription. Furthermore, CLOCK binds to *Dbp* promoter in a similar fashion (Figure S1), [27]. This also suggests that CLOCK binds to *C/ebpα* promoter in a circadian manner.

**Figure 5:**
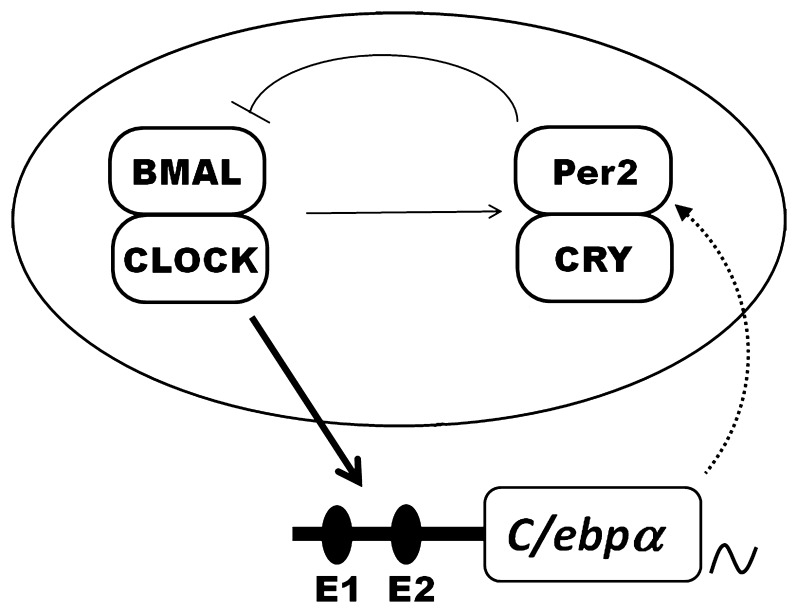
Links between *C/ebpα* and *Clock* gene expressions. An interactive feedback loop between *C/ebpα* and the core clock genes feedback loop is indicated. The core clock genes feedback loop is enclosed in an oval. Transcriptional activators CLOCK and BMAL1 drive *Per2* and *C/ebpα* gene expression. Per and CRY proteins inhibit CLOCK/BMAL1 activity. Thick line denotes regulation based on present findings. Dotted arrow comes from other papers [20], [21].

Our findings suggest that *C/ebpα* is one of clock-controlled genes regulated by the circadian negative feedback (Oval in Figure 5). Interestingly, others have reported that the *Per2* promoter region has several potential *C/ebpα* binding sites to activate *Per2* transcription [19], [20] (Dotted line in Figure 5). Furthermore, previous papers indicate that *Per2* and *C/ebpα* have the same effects for tumor suppression events [19], [20]. Thus, we propose an interactive feedback loop between the negative feedback loop and *C/ebpα* rhythmic expression (Figure 5). Recently, the circadian expression of the gene encoding glycogen synthase was found to be damped in livers of *C/ebpα* conditional-knockout mice [33]. These data support that glucose metabolism might be involved in an interactive feedback loop in livers.

Recent studies have revealed an association between the circadian clock disruption and metabolic syndrome [28], [33]–[35]. The *C/ebpα* gene has divergent roles and functions, especially in glucose metabolism [10], [35], [36]. These findings also suggest close links between the circadian clock-controlled gene, *C/ebpα* and metabolic activity. We propose that disrupted clock genes expression may cause metabolic syndrome through deregulation of *C/ebpα*.

## Supporting Information

Figure S1 **ChIP assay for CLOCK binding to** ***Dbp*** **promoter.** Binding activity between CLOCK and E box element in *Dbp* (*D site albumin promoter binding protein*) promoter sequence was evaluated to confirm the validity of our ChIP assay. Experiment was performed as described in the materials and methods. Each time point comes from triplicate experiments. Data are presented as mean ± SEM (n = 3, *: p = 0.01).
(TIF)
